# Human natural killer cells exhibit potent antifungal activity against azole-resistant *Aspergillus fumigatus* and diverse filamentous fungi

**DOI:** 10.1128/spectrum.03372-25

**Published:** 2026-04-21

**Authors:** Hotaka Namie, Takahiro Takazono, Satoshi Irifune, Satoru Koga, Yuya Ito, Nana Nakada, Tatsuro Hirayama, Masataka Yoshida, Kazuaki Takeda, Shotaro Ide, Naoki Iwanaga, Masato Tashiro, Naoki Hosogaya, Noriho Sakamoto, Haruki Okamura, Yoshimasa Tanaka, Katsunori Yanagihara, Tomoya Nishino, Hiroshi Mukae, Koichi Izumikawa

**Affiliations:** 1 Department of Infectious Diseases, Nagasaki University Graduate School of Biomedical Sciences 200674 , Nagasaki City, Nagasaki, Japan; 2 Department of Respiratory Medicine, Nagasaki University Hospital 88380, Nagasaki City, Nagasaki, Japan; 3 Japanese Red Cross Nagasaki Genbaku Isahaya Hospital, Isahaya city, Nagasaki, Japan; 4 Health Center, Nagasaki University 12961, Nagasaki City, Nagasaki, Japan; 5 Department of Pharmacotherapeutics, Nagasaki University Graduate School of Biomedical Sciences 200674 , Nagasaki City, Nagasaki, Japan; 6 Infectious Diseases Experts Training Center, Nagasaki University Hospital 88380, Nagasaki City, Nagasaki, Japan; 7 Department of Infectious Disease Medicine, Yokohama City University Graduate School of Medicine 13155, Yokohama City, Kanagawa, Japan; 8 Clinical Research Center, Nagasaki University Hospital 88380, Nagasaki City, Nagasaki, Japan; 9 Laboratory of Tumor Immunology and Cell Therapy, Hyogo College of Medicine 12818, Nishinomiya, Japan; 10 Center for Medical Innovation, Nagasaki University 12961, Nagasaki City, Nagasaki, Japan; 11 Department of Laboratory Medicine, Nagasaki University Hospital 88380, Nagasaki City, Nagasaki, Japan; 12 Department of Nephrology, Nagasaki University Hospital 88380, Nagasaki City, Nagasaki, Japan; 13 Infection Control and Education Center, Nagasaki University Hospital 88380, Nagasaki City, Nagasaki, Japan; Virginia-Maryland College of Veterinary Medicine, Blacksburg, Virginia, USA

**Keywords:** azole resistance, filamentous fungi, interferon-γ, invasive aspergillosis, NK cell, XTT assay

## Abstract

Invasive aspergillosis in immunocompromised individuals is associated with a poor prognosis. Recently, the global emergence of azole-resistant *Aspergillus fumigatus* has underscored the urgent need for novel therapeutic strategies beyond conventional small-molecule antifungal agents. Natural killer (NK) cells, which are characterized by the absence of CD3 and presence of CD56 surface markers, play a pivotal role in the immune response against fungal pathogens and in the elimination of malignant cells and virus-infected host cells. Although the antifungal activity of NK cells against *Aspergillus* spp. and other fungi has been demonstrated, the efficacy of *ex vivo*-expanded human NK cells against azole-resistant *A. fumigatus* has not been investigated. NK cells, which were expanded from the CD3^−^ peripheral blood mononuclear cells obtained from healthy donors, were co-cultured with fungi. Antifungal activity was assessed using the 2,3-(bis2-methoxy-4-nitro-5-sulfophenyl)-2H-tetrazolium-5-carboxyanilide reduction assay. NK cells demonstrated antifungal activity against azole-resistant *A. fumigatus* and other filamentous fungi. These findings suggest that a direct interaction between NK cells and hyphae is critical for exerting antifungal effects, with degranulation likely contributing to the underlying mechanism. However, contrary to previous reports, we failed to observe the direct antifungal activity of interferon-γ secreted by NK cells under our experimental conditions. Cellular immunotherapy using NK cells may represent a promising therapeutic approach for the treatment of azole-resistant aspergillosis.

**IMPORTANCE:**

*Aspergillus fumigatus* is a ubiquitous fungus that causes invasive aspergillosis (IA) in immunocompromised patients, with poor prognosis. Furthermore, azole-resistant *A. fumigatus* has spread globally in recent years. The present antifungal drugs pose challenges such as drug resistance and adverse events; thus, the development of novel therapies for IA is needed beyond the antifungal drugs. In cases of IA in immunocompromised patients with poor response to antifungal drugs, cellular immunotherapy is a reasonable approach. This study demonstrated that human natural killer (NK) cells derived from peripheral blood exhibit antifungal activity against azole-resistant *A. fumigatus* comparable to that of azole-susceptible strain. This indicates that cellular immunotherapy using NK cells could become an effective therapy for IA caused by azole-resistant *A. fumigatus*. Investigating the mechanism by which NK cells exhibit antifungal activity against *A. fumigatus* could lead to the development of novel therapies for IA.

## INTRODUCTION

*Aspergillus* spp. are ubiquitous environmental fungi, with *Aspergillus fumigatus* being the most common cause of invasive aspergillosis (IA) in immunocompromised individuals. Despite the availability of standard antifungal therapies, the prognosis for IA remains poor, with reported mortality rates of approximately 30%–60% (1–4). Azole-resistant *A. fumigatus* has emerged globally over recent years (5–7), prompting its designation as the highest-priority fungal pathogen in the World Health Organization’s 2022 Fungal Priority Pathogen List. A previous study reported that the 90-day mortality rate of IA patients infected with azole-resistant strains was approximately 25% higher than that of IA patients infected with azole-sensitive strains (8). Moreover, adverse effects associated with antifungal agents represent a significant clinical concern (9), highlighting the urgent need for novel therapeutic modalities beyond conventional small-molecule compounds.

The host immune response to *Aspergillus* spp. is primarily mediated by innate immune cells, such as alveolar macrophages and neutrophils (10), as evidenced by the increased risk of IA in neutropenic patients. However, recent studies have demonstrated that both innate immune cells—including natural killer (NK) cells and Vγ9Vδ2 T cells—and adaptive immune cells, such as T lymphocytes, play critical roles in antifungal defense (11–13).

NK cells are large granular lymphocytes characterized by CD3 absence and CD56 expression, and they exert cytotoxic activity as part of the innate immune system. Their primary functions include the elimination of tumor cells and virus-infected cells. Cellular immunotherapy using allogeneic NK cell therapies is currently under development as a novel cancer treatment, offering advantages such as a reduced risk of graft-versus-host disease and cytokine release syndrome compared to T cell-based therapies (14). Clinical trials have demonstrated the efficacy and safety of allogeneic NK cell therapies and chimeric antigen receptor (CAR)-NK cells in both hematologic malignancies and solid tumors (15–18).

Beyond their role in tumor immunity, NK cells are increasingly recognized for their involvement in infectious diseases. In a murine model of invasive pulmonary aspergillosis, NK cell deficiency was associated with an increased fungal burden in the lungs, suggesting the protective role of NK cells in antifungal immunity (19). *In vitro* studies have also reported the antifungal activity of NK cells against *Mucor* spp., *Cryptococcus* spp., *Candida* spp., and *Aspergillus* spp. (20–23). However, to date, no studies have specifically examined the antifungal effects of *ex vivo*-expanded human NK cells against azole-resistant *A. fumigatus*. Furthermore, mechanisms underlying NK cell-mediated antifungal activity remain incompletely understood. Although interferon gamma (IFN-γ) released by NK cells has been proposed to exert direct antifungal effects (24), the precise molecular mechanisms involved have yet to be elucidated.

This study aimed to evaluate the antifungal activity of NK cells expanded from human peripheral blood against azole-resistant *A. fumigatus* and to characterize the immune responses of NK cells to *Aspergillus* spp., with the goal of developing novel immunotherapeutic strategies.

## MATERIALS AND METHODS

### Preparation of NK and Vγ9Vδ2 T cells

Peripheral blood samples were obtained from healthy volunteers, from which innate immune cells, NK cells, and Vγ9Vδ2 T cells were subsequently expanded. To derive NK cells, CD3^−^ peripheral blood mononuclear cells were cultured for 10 days in the presence of interleukin (IL)-2 and IL-18 (25). Vγ9Vδ2 T cells were stimulated with tetrakis pivaloyloxymethyl 2-(thiazole-2-ylamino) ethylidene-1,1-bisphosphonate (PTA) and expanded over 11 days in the presence of IL-2. Detailed protocols for cell expansion are provided in the Supplementary material (Fig. S1A, Fig. S1B, Fig. S1C and Fig. S2).

To assess donor variability and reproducibility, NK cell-based experiments were conducted using blood samples from at least three independent donors. All participants provided informed consent prior to sample collection.

### Fungal strains

All filamentous fungi were cultured on potato dextrose agar at 37°C for 4–7 days. Conidia and hyphae were harvested and suspended in 1% Tween 20 (FUJIFILM Wako Pure Chemical Corp., Chuo-ku, Osaka, Japan) in phosphate-buffered saline (PBS). Hyphae were removed using a 0.4 µm cell strainer, and the resulting conidial suspension was centrifuged at 3,000 rpm for 5 min, washed once with PBS, and resuspended in RPMI 1640 medium (FUJIFILM Wako Pure Chemical Corp.). The conidial concentration was adjusted to 1.0 × 10⁶ conidia/mL and dispensed into 48-well plates (150 µL/well) and 24-well plates (300 µL/well). Fungal suspensions were incubated statically at 37°C in 5% CO₂ for 24 h. Owing to its slower growth rate, *Aspergillus terreus* was incubated for 96 h prior to the 2,3-bis(2-methoxy-4-nitro-5-sulfophenyl)-2H-tetrazolium-5-carboxyanilide (XTT) assay.

*A. fumigatus* strain Af293 was used as the azole-sensitive reference strain. Azole-resistant strains included NGS-ER7, IFM63240, and IFM64258. NGS-ER7 is an environmental isolate from Europe exhibiting azole resistance due to mutations in *cyp51A* (Y121F and T289) and *hmg1* (E105K and T289) (minimum inhibitory concentrations [MICs], Clinical and Laboratory Standards Institute M38: itraconazole, 2 µg/mL; voriconazole, >8 µg/mL) (26). The IFM63240 and IFM64258 clinical isolates were provided by Chiba University, Japan, with azole resistance likely mediated by mutations outside *cyp51A*. IFM63240 (MICs: itraconazole > 8 µg/mL; voriconazole = 4 µg/mL) harbors S269F in *hmg1* and A350T in *erg6*, whereas IFM64258 (MICs: itraconazole = 4 µg/mL; voriconazole = 8 µg/mL) harbors F390Y in *hmg1* and V206A in *erg6* (27).

Other *Aspergillus* spp. used in this study included *Aspergillus flavus* (NBRC6343), *Aspergillus niger* (NBRC10564), and *Aspergillus terreus* (NBRC6346). *Mucorales* species included *Rhizopus oryzae* (IFM46105), *Cunninghamella bertholletiae* (IFM59518), *Rhizopus microsporus* (IFM46417), *Mucor circinelloides* (IFM55051), and *Rhizopus pusillus* (IFM40783).

### Evaluation of antifungal effect

The antifungal activity of innate immune cells against filamentous fungi was evaluated using the XTT assay kit (Merck & Co., Darmstadt, Hesse, Germany), as previously described (13). The XTT assay is a widely utilized method for assessing the metabolic activity of bacteria and filamentous fungi by measuring optical density (OD), thereby reflecting the viability of living cells (28, 29).

NK cells and/or Vγ9Vδ2 T cells were adjusted to a concentration of 2 × 10⁷ cells/mL and co-cultured with fungi at effector cell to conidia ratio of 100:3. The co-cultures were incubated at 37°C in 5% CO₂ for 24 h. Following incubation, cells were lysed with ice-cold distilled water for 30 min. The supernatants were removed, and XTT reagent was added to each well at a final concentration of 0.3 mg/mL. Plates were incubated in the dark at 37°C for 16 h. Subsequently, 100 μL supernatant was transferred to a 96-well plate, and the OD was measured at 450 and 620 nm. Antifungal activity was calculated using the following formula: antifungal effect (%) = (1 − *X*/*Y*) × 100, where *X* represents the OD value under experimental conditions (including innate immune cells and/or antifungal agents), and *Y* represents the OD value of the fungus-only negative control. Antifungal assays against *Aspergillus* spp. and *Mucor* spp. were performed in 48-well plates, while all other experiments were conducted in 24-well plates.

To examine whether direct contact between innate immune cells and fungal hyphae is necessary for antifungal activity, cell culture inserts (Merck KGaA, Darmstadt, Germany) were employed. These inserts allow the diffusion of soluble factors while preventing physical contact between immune cells and hyphae adhered to the bottom of the well. Experiments were conducted under the following conditions: fungus alone (negative control), NK cells + fungus (direct contact), and NK cells in insert + fungus (no contact). Ethylene glycol tetraacetic acid (EGTA), a calcium chelator known to inhibit degranulation processes such as perforin release, was used to assess the role of degranulation in antifungal activity. Prior to co-culture, the immune cells were pretreated with RPMI medium containing 5 mM EGTA for 30 min at 25°C. Calcium chloride (CaCl_2_) at an equivalent concentration was used to reverse the chelating effect of EGTA.

To evaluate the antifungal activity of soluble factors, supernatants from prior co-cultures were collected, filtered through a 0.22 μm membrane, and stored at −80°C until use in the XTT assay. Recombinant interferon-γ (rIFN-γ; PeproTech, Inc., Cranbury, NJ, USA) was tested at concentrations of 31.25–3,000 pg/mL in RPMI 1640 medium.

### Cytokine assay

A suspension of *A. fumigatus* conidia was adjusted to 1 × 10^7^ conidia/mL, and 50 μL aliquots were dispensed into 96-well round-bottom plates. Plates were incubated at 37°C in a humidified atmosphere containing 5% CO₂ for 24 h. For co-culture experiments, the supernatant was carefully removed, and 100 μL NK cell suspension, adjusted to 1 × 10^7^ cells/mL, was added to each well. As a control, the same volume of the NK cell suspension was added to wells without fungal conidia. Subsequently, 100 μL RPMI 1640 medium supplemented with IL-2 was added to achieve a final IL-2 concentration of 1,000 IU/mL. NK cells were cultured for 6 to 24 h. At each time point, plates were centrifuged at 1,700 rpm for 5 min, and 150 μL supernatant was collected and stored at −80°C until further analysis.

The concentration of IFN-γ in supernatants from NK cells and *A. fumigatus* co-cultures was quantified using a human IFN-γ enzyme-linked immunosorbent assay (ELISA) kit (PeproTech, Cranbury, NJ, USA). Briefly, 96-well flat-bottom plates (Thermo Fisher Scientific, Waltham, MA, USA) were coated overnight at 25°C with 1 μg/mL of human IFN-γ capture antibody diluted in PBS. Wells were washed four times with 300 μL of PBS containing 0.05% Tween 20 and subsequently blocked with 300 μL of PBS containing 1% bovine serum albumin (BSA). After another washing step, 100 μL of each sample was added to each well and incubated at 4°C for 24 h. Wells were washed again and incubated with 100 μL of biotin-conjugated anti-human IFN-γ antibody (0.25 μg/mL in PBS containing 0.05% Tween 20 and 0.1% BSA) for 2 h at 25°C. Following additional washes, 100 μL horseradish peroxidase-conjugated avidin solution was added and incubated for 30 min at 25°C. After the final wash, 100 μL 2,2′-azino-bis(3-ethylbenzothiazoline-6-sulfonic acid) diammonium salt substrate solution (Merck KGaA) was added and incubated for 5 min at 25°C. The reaction was terminated with 1% sodium dodecyl sulfate, and the OD was measured at 405 and 620 nm.

### Statistical analysis

Statistical analyses were performed using the GraphPad Prism software (version 9.3.1; GraphPad Software, San Diego, CA, USA). Differences between two groups were assessed using a two-tailed Student’s *t*-test, while comparisons among multiple groups were evaluated using one-way or two-way analysis of variance, followed by Tukey’s *post hoc* test, where appropriate. Experiments were performed using cells derived from a single donor, with multiple wells treated as technical replicates per condition. To assess reproducibility, experiments were also independently repeated at least three times using cells from different donors.

## RESULTS

### Human NK cells exhibit antifungal activity against *A. fumigatus*

**Fig 1:**
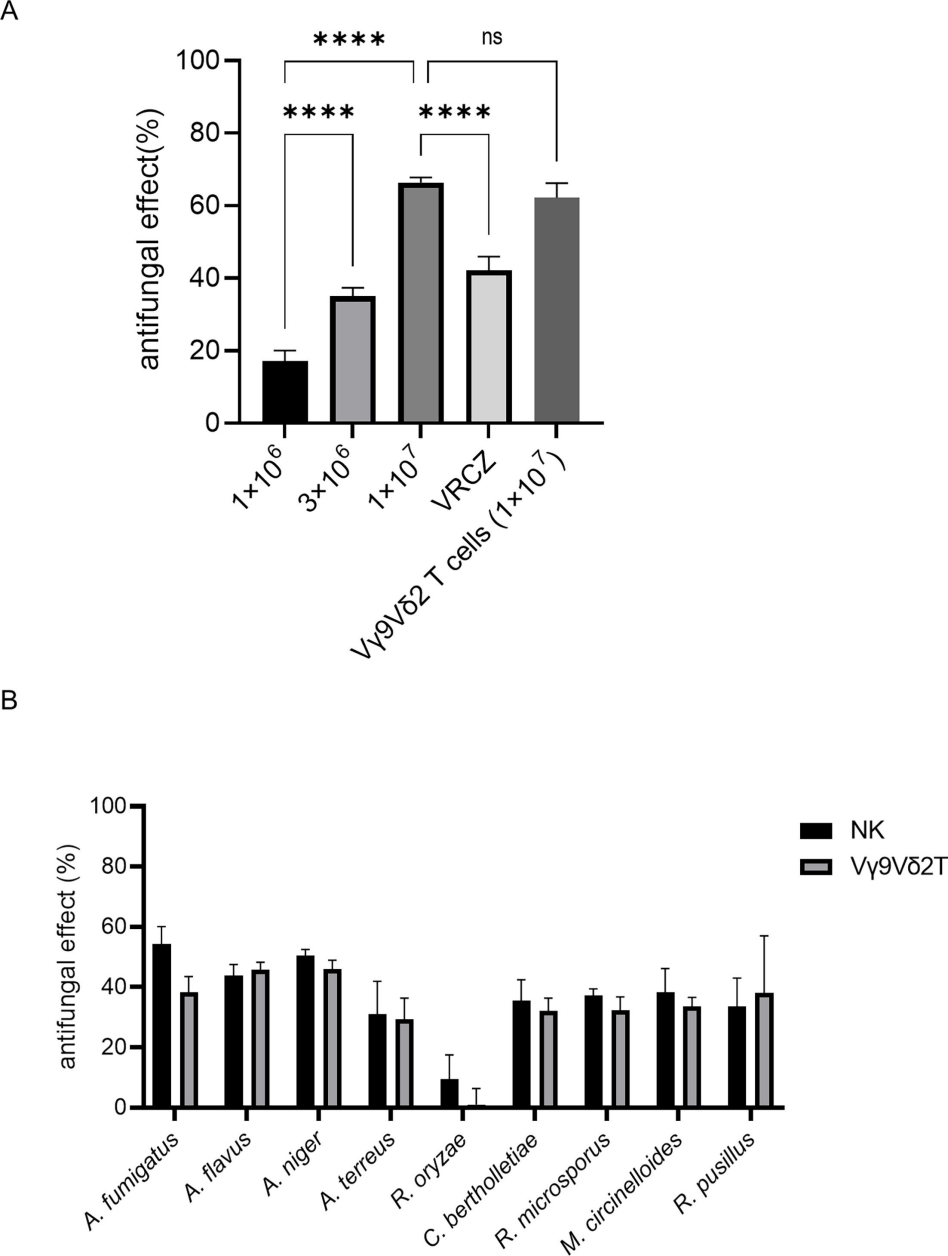
Antifungal activities of natural killer (NK) cells and Vγ9Vδ2 T cells against filamentous fungi. (**A**) Dose-dependent antifungal effects of NK cells against *Aspergillus fumigatus*, as assessed by 2,3-(bis2-methoxy-4-nitro-5-sulfophenyl)-2H-tetrazolium-5-carboxyanilide (XTT) assay (****P < 0.0001; ns, not significant). Vγ9Vδ2 T cells and voriconazole (VRCZ) at 2 μg/mL were used as positive controls. Bar graph shows the mean and standard deviation of technical replicates (*n* = 8 for NK cells; *n* = 6 for Vγ9Vδ2 T cells and VRCZ). (**B**) Antifungal activities of NK cells and Vγ9Vδ2 T cells against various *Aspergillus* spp. (*Aspergillus flavus*, *Aspergillus niger*, and *Aspergillus terreus*) and mucormycetes (*Rhizopus oryzae*, *Cunninghamella bertholletiae*, *Rhizomucor microsporus*, *Mucor circinelloides*, and *Rhizopus pusillus*). Bar graph shows the mean and standard deviation of technical replicates (*n* = 4). Data are representative of at least three independent experiments.

To assess the antifungal activity of human NK cells against filamentous fungi, an XTT reduction assay was performed. Human Vγ9Vδ2 T cells, previously demonstrated to possess antifungal properties, were included as positive controls (13). As shown in Fig. 1A, NK cells exhibited concentration-dependent antifungal effects against *A. fumigatus*. Notably, at a concentration of 1 × 10^7^ cells/well (effector-to-target ratio of 100:3), the antifungal activity of NK cells surpassed that of voriconazole. The culture supernatant from high-concentration NK cells (1 × 10^7^ cells per well) cultured for 24 h without fungi showed no antifungal activity (Fig. S3). This result indicates that changes in pH and nutrients associated with high-concentration cell culture did not affect the metabolic activity of *A. fumigatus*.

Furthermore, NK cells demonstrated comparable antifungal effects against other *Aspergillus* spp. and members of the *Mucorales* order (Fig. 1B). In contrast, both NK cells and Vγ9Vδ2 T cells exhibited relatively lower antifungal activity against *Rhizopus oryzae* than that against other filamentous fungi.

### Human NK cells exhibit antifungal activity against azole-resistant *A. fumigatus*

**Fig 2:**
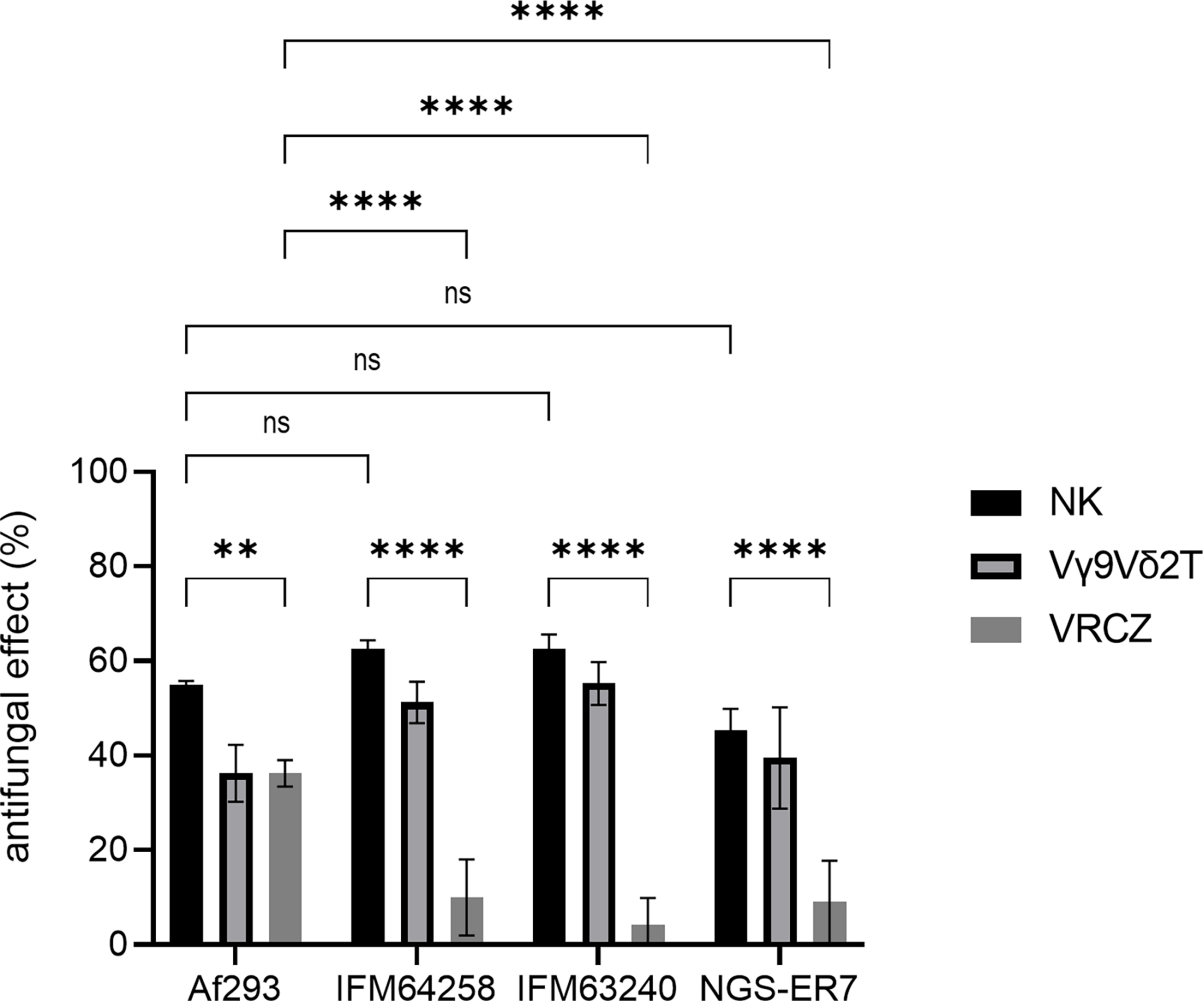
Antifungal activity of natural killer (NK) and Vγ9Vδ2 T cells against the wild-type and azole-resistant *Aspergillus fumigatus* strains. NK cells and Vγ9Vδ2 T cells voriconazole (VRCZ) at 1 μg/mL were evaluated for their antifungal effects against the wild-type *A. fumigatus* strain Af293 and azole-resistant strains IFM64258, IFM43240, and NGS-ER7. Statistically significant differences are indicated as follows: **P = 0.003, ****P < 0.0001 and ns, not significant. Bar graph shows the mean and standard deviation of technical replicates (*n* = 4). Data are representative of at least three independent experiments.

To determine the antifungal activity of human NK cells against azole-resistant *A. fumigatus*, we employed the same XTT-based assay system used for azole-susceptible strains. As illustrated in Fig. 2, NK cells demonstrated antifungal effects against azole-resistant *A. fumigatus*, comparable to those observed against azole-susceptible strains. In contrast, voriconazole exhibited potent antifungal activity against azole-susceptible strains but showed markedly reduced efficacy against azole-resistant isolates. As demonstrated in a prior study (13), Vγ9Vδ2 T cells exhibited antifungal activity against azole-resistant strains, comparable to or higher than those observed against azole-susceptible strains.

### Direct contact between NK cells and hyphae is essential for antifungal activities

**Fig 3:**
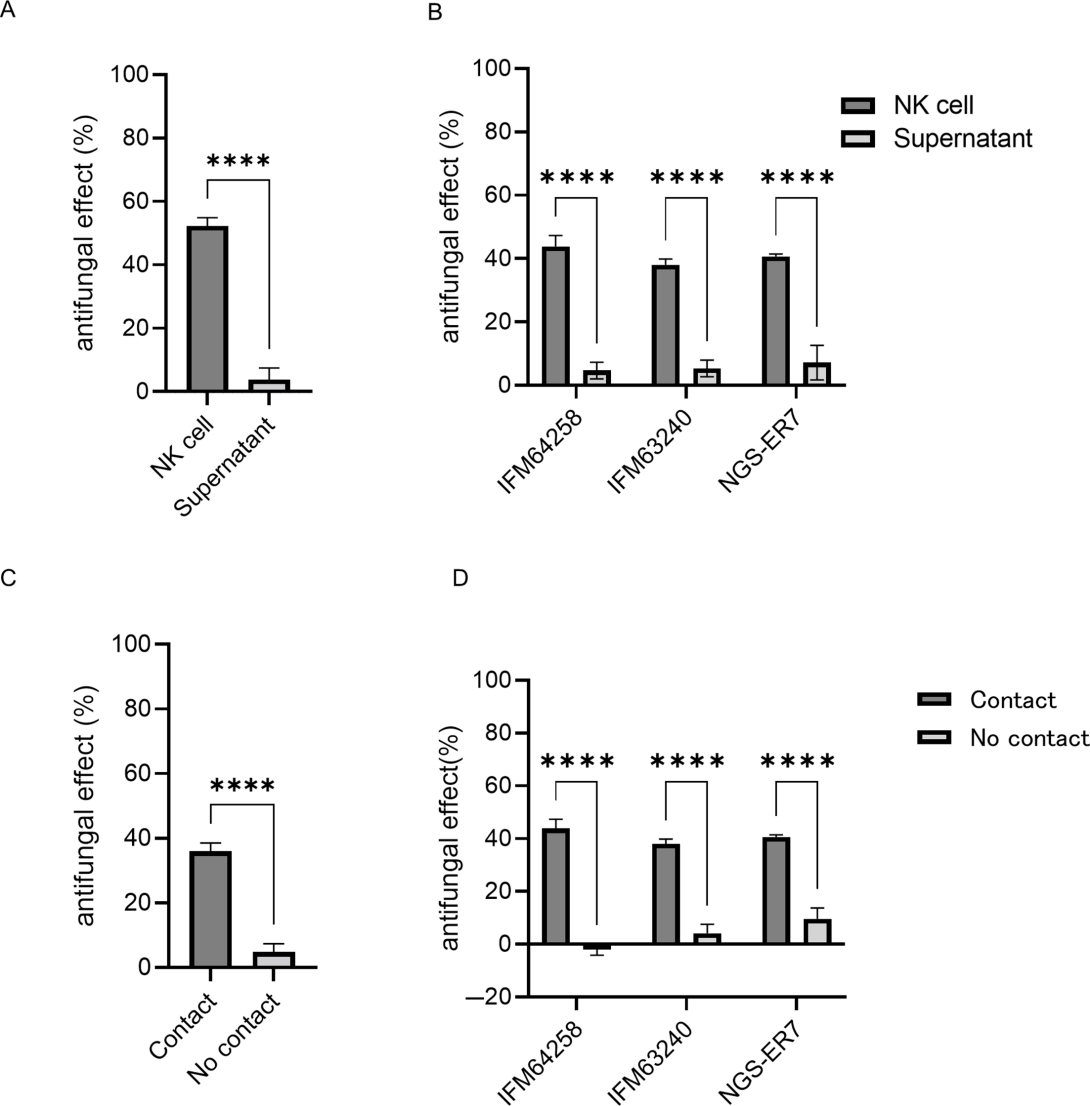
Antifungal activity of IL-2/IL-18-expanded natural killer (NK) cells and co-culture supernatants under non-contact conditions. (**A**) Antifungal effects of NK cells and the supernatant collected after 24-h co-culture of NK cells with azole-susceptible *Aspergillus fumigatus* (Af293) hyphae (****P < 0.0001). Bar graph shows the mean and standard deviation of technical replicates (*n* = 4). (**B**) Antifungal effects of NK cells and the supernatant collected after 24-h co-culture of NK cells with azole-resistant *A. fumigatus* (IFM64258, IFM63240, and NGS-ER7) hyphae (****P < 0.0001). Bar graph shows the mean and standard deviation of technical replicates (*n* = 6). (**C**) NK cells and azole-susceptible *A. fumigatus* (Af293) hyphae were cultured using a cell insert system to prevent direct cell-to-hyphae contact, and antifungal activity was assessed (****P < 0.0001). Bar graph shows the mean and standard deviation of technical replicates (*n* = 4). (**D**) NK cells and azole-resistant *A. fumigatus* hyphae (IFM64258, IFM63240, and NGS-ER7) were cultured using a cell insert system to prevent direct cell-to-hyphae contact, and antifungal activity was assessed (****P < 0.0001). Bar graph shows the mean and standard deviation of technical replicates (*n* = 6). Data are representative of at least three independent experiments.

To elucidate the mechanism underlying the antifungal activity of NK cells against *A. fumigatus*, we first assessed the effect of supernatants derived from NK cell-*A*. *fumigatus* co-cultures on hyphal viability. As shown in Fig. 3A and B, the supernatants did not exhibit any detectable antifungal activity. This finding does not exclude the possibility that soluble effector molecules may be structurally unstable or that they rapidly degraded during the freeze-thaw process.

To further examine the requirement for direct cell-fungus interaction, we employed a transwell culture system that physically separates NK cells from fungal hyphae while allowing the exchange of soluble factors. Under these conditions, NK cells failed to exert antifungal effects (Fig. 3C and D), indicating that direct contact between NK cells and fungal hyphae is critical for antifungal activity.

### Degranulation is a mechanism underlying antifungal activities of NK cells

**Fig 4:**
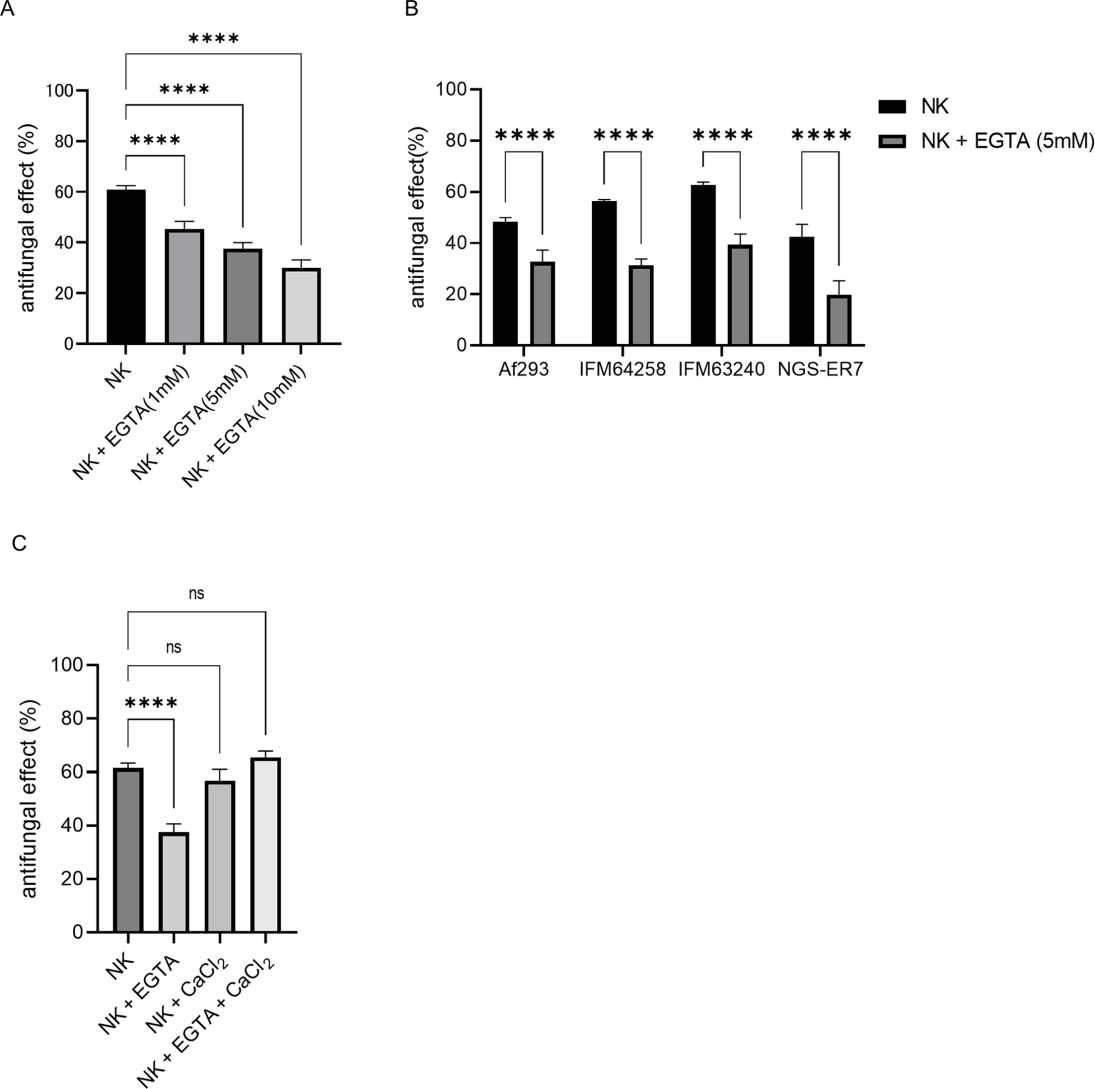
Involvement of degranulation in the antifungal activity of natural killer (NK) cells against *Aspergillus fumigatus*. (**A**) Antifungal effect of NK cells against azole-susceptible *A. fumigatus* (Af293) hyphae with different concentrations of ethylene glycol tetraacetic acid (EGTA), which is an inhibitor of degranulation. The antifungal effect was determined by treating NK cells with EGTA at concentrations of 1, 5, and 10 mM for 30 min before co-culture (****P < 0.0001). Bar graph shows the mean and standard deviation of technical replicates (*n* = 6). (**B**) Antifungal effect of NK cells against azole-resistant *A. fumigatus* (IFM64258, IFM63240, and NGS-ER7) hyphae with EGTA. The antifungal effect was determined by treating NK cells with EGTA at concentrations of 5 mM for 30 min before co-culture (***P < 0.0001). Bar graph shows the mean and standard deviation of technical replicates (*n* = 6). (**C**) Antifungal effect of NK cells against azole-susceptible *A. fumigatus* (Af293) with EGTA, calcium chloride (CaCl_2_), or both. EGTA and CaCl_2_ were used at concentrations of 5 mM (****P < 0.0001; ns, not significant ). Bar graph shows the mean and standard deviation of technical replicates (*n* = 4). Data are representative of at least three independent experiments.

To analyze the involvement of degranulation in the antifungal activity of NK cells, we utilized EGTA, a calcium-specific chelating agent that is commonly employed to inhibit degranulation in NK cells and cytotoxic T lymphocytes. Pretreatment of NK cells with EGTA prior to co-culture with *A. fumigatus* resulted in a concentration-dependent reduction in the antifungal activity (Fig. 4A). Furthermore, the antifungal activity of NK cells against azole-resistant *A. fumigatus* was also reduced by EGTA treatment (Fig. 4B). Notably, the addition of CaCl₂ to the culture medium, which competes with EGTA for calcium binding, restored the antifungal activity of NK cells (Fig. 4C). These findings suggest that calcium-dependent degranulation is a critical mechanism by which NK cells exert antifungal effects against *A. fumigatus*. No cytotoxicity was observed in the cell viability assessment using trypan blue staining 24 h after exposure to 5 mM EGTA (data not shown).

### IFN-γ does not exhibit direct antifungal activity against *A. fumigatus*

**Fig 5:**
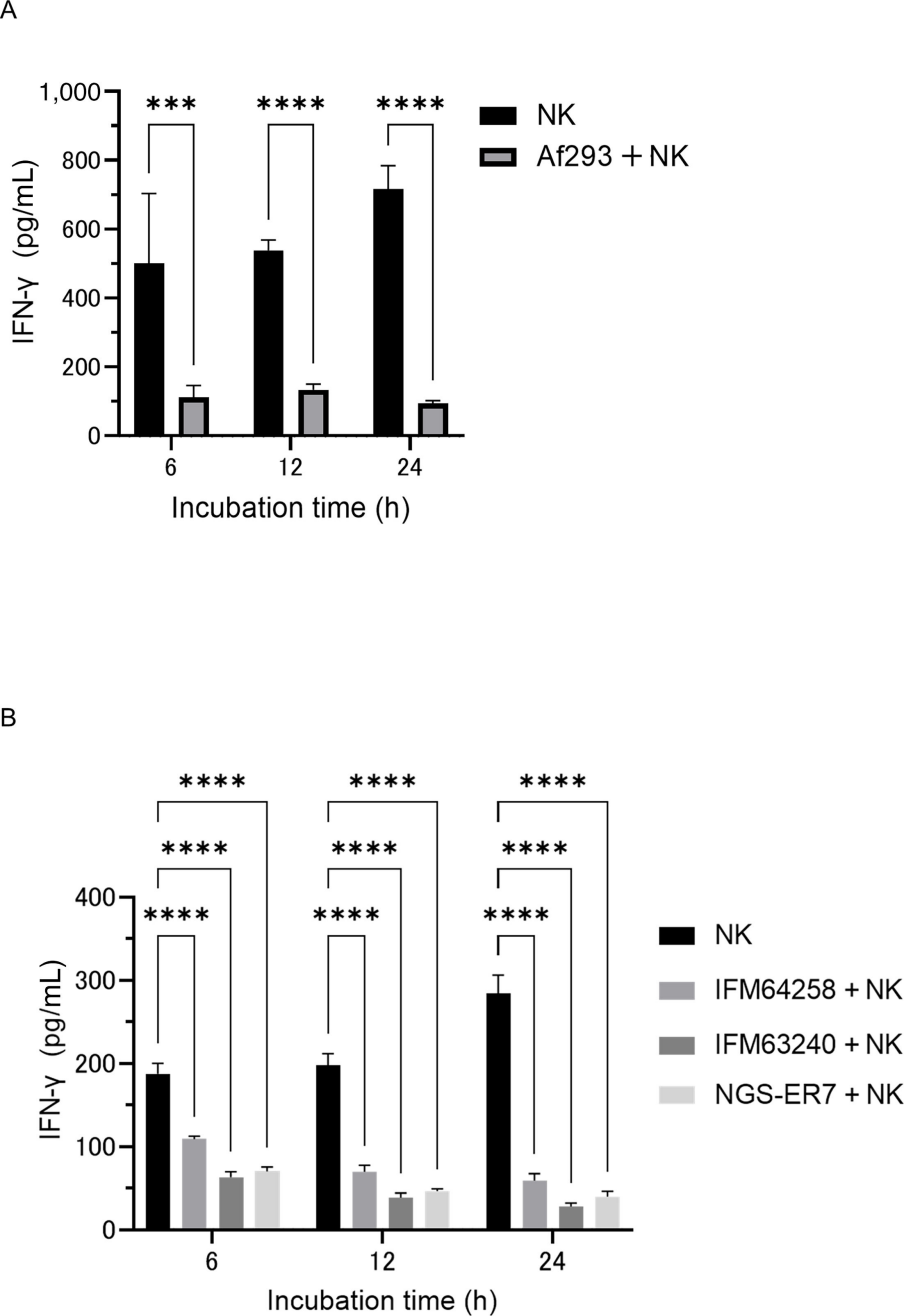
Effects of *Aspergillus fumigatus* hyphae on interferon gamma (IFN-γ) secretion by IL-2/IL-18-expanded natural killer (NK) cells. (**A**) Interferon-γ concentration in the supernatant after co-culturing NK cells and azole-susceptible *A. fumigatus* (Af293). Co-culture with hyphae significantly reduced IFN-γ release (***P = 0.001, ****P < 0.0001). Bar graph shows the mean and standard deviation of technical replicates (*n* = 4). (**B**) Interferon-γ concentration in the supernatant after co-culturing NK cells and azole-resistant *A. fumigatus* (IFM64258, IFM63240, and NGS-ER7). Co-culture with hyphae significantly reduced IFN-γ release (****P < 0.0001). Bar graph shows the mean and standard deviation of technical replicates (*n* = 4). IFN-γ in the culture supernatant was measured at multiple time points under two culture conditions: NK cells cultured alone and NK cells co-cultured with *A. fumigatus* hyphae. Data are representative of at least three independent experiments.

Previous studies have suggested that IFN-γ exerts direct antifungal effects against *A. fumigatus* (24, 30). To confirm this finding, we first quantified IFN-γ secretion from NK cells using an ELISA. As shown in Fig. 5A and B, IFN-γ levels were reduced when NK cells were co-cultured with *A. fumigatus*.

**Fig 6:**
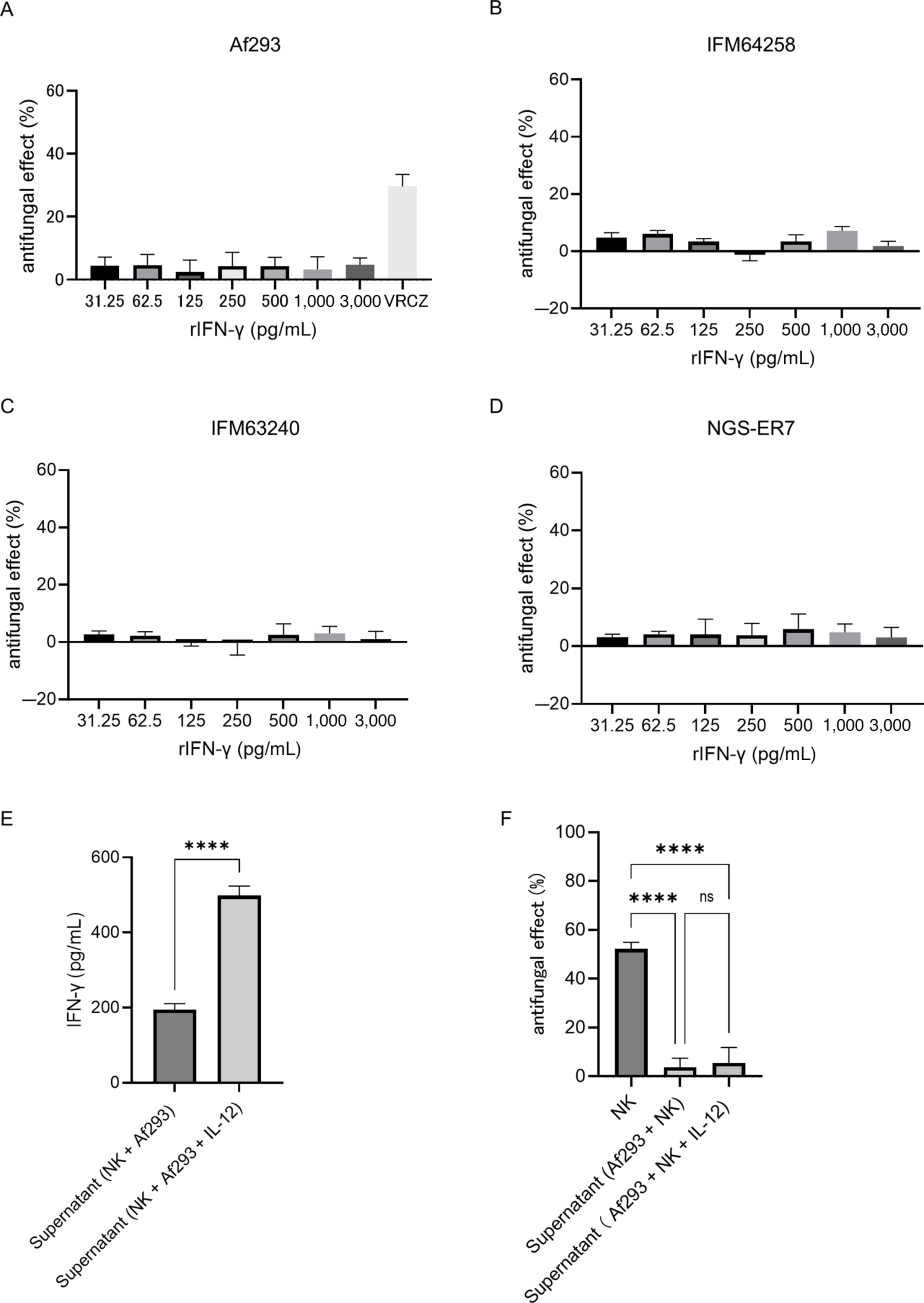
Direct antifungal activity of IFN-γ against *Aspergillus fumigatus*. (**A**) Direct antifungal effects of recombinant interferon gamma (rIFN-γ) against azole-susceptible *A. fumigatus* (Af293). Voriconazole (VRCZ) at 2 μg/mL was used as a positive control. Bar graph shows the mean and standard deviation of technical replicates (*n* = 7). (**B**) Direct antifungal activity of rIFN-γ against azole-resistant *A. fumigatus* (IFM64258). Bar graph shows the mean and standard deviation of technical replicates (*n* = 6). (**C**) Direct antifungal activity of rIFN-γ against azole-resistant *A. fumigatus* (IFM63240). Bar graph shows the mean and standard deviation of technical replicates (*n* = 6). (**D**) Direct antifungal activity of rIFN-γ against azole-resistant *A. fumigatus* (IFM63240). Bar graph shows the mean and standard deviation of technical replicates (*n* = 6). (**E**) IFN-γ levels in the supernatant following the co-culture of natural killer (NK) cells with azole-susceptible *A. fumigatus* hyphae. NK cells were stimulated with interleukin-12 (IL-12) at 10 ng/mL (****P < 0.0001). Bar graph shows the mean and standard deviation of technical replicates (*n* = 4). (**F**) Antifungal activity of co-culture supernatants containing IFN-γ released from NK cells against azole-susceptible *A. fumigatus* (****P < 0.0001; ns, not significant ). Bar graph shows the mean and standard deviation of technical replicates (*n* = 4). Data are representative of at least three independent experiments.

Next, we assessed the antifungal activity of rIFN-γ using the XTT assay. As shown in Fig. 6A through D, rIFN-γ did not exhibit direct antifungal effects against *A. fumigatus*. To further evaluate the role of IFN-γ, NK cells were stimulated with interleukin-12 (IL-12) at 10 ng/mL, and the resulting co-culture supernatants—containing elevated levels of IFN-γ—were tested for antifungal activity. Although IL-12 stimulation significantly increased IFN-γ concentrations in the supernatants (Fig. 6E), no enhancement in antifungal activity was observed compared to unstimulated conditions (data not shown). Moreover, supernatants from IL-12–stimulated co-cultures failed to exert direct antifungal effects similar to those from unstimulated co-cultures (Fig. 6F).

These findings indicate that IFN-γ, despite its immunomodulatory role, does not directly contribute to the antifungal activity of NK cells against *A. fumigatus* under the tested conditions.

## DISCUSSION

Herein, we examined the antifungal activity of *in vitro*-expanded human NK cells against filamentous fungi using the XTT assay and explored the mechanisms underlying NK cell-mediated antifungal effects. Our findings demonstrated that IL-2/IL-18-expanded NK cells exert significant antifungal activity against a broad spectrum of filamentous fungi, including *A. fumigatus* and azole-resistant *A. fumigatus*. These results suggest that direct contact between NK cells and fungal hyphae is essential for antifungal efficacy and that degranulation is a key mechanism involved. Notably, IFN-γ did not exhibit direct antifungal effects, in contrast to the findings of previous reports that implicated IFN-γ as a critical mediator of NK cell antifungal activity.

Antifungal properties of NK cells against various fungal pathogens have been documented in previous studies, including *in vitro* activity against *Rhizopus oryzae* and other mucoromycetes (23, 31), *Candida albicans* (21), and *Cryptococcus neoformans* (20, 22). Azole antifungals remain the cornerstone of aspergillosis treatment (32), primarily by inhibiting ergosterol synthesis, which is a vital component of the fungal cell membrane. Mechanisms of azole resistance include reduced binding affinity to the target enzyme Cyp51A (33), Cyp51A overexpression (34), and efflux pump activation (35). Recent evidence suggests additional resistance mechanisms involving genetic mutations beyond Cyp51A (27), underscoring the need for alternative therapeutic strategies. Importantly, our data indicated that NK cell-mediated antifungal activity is not compromised by these resistance pathways.

In this study, the antifungal activity of NK cells against *R. oryzae* was lower compared to other *Mucor* spp. Conversely, previous research has reported that NK cells exhibit sufficient antifungal activity against *Mucor* spp., including *R. oryzae* (23, 31), which contradicts our study. Differences in the NK cell stimulation, the fungal strains, and XTT assay conditions may have led to these conflicting results.

Previous studies have proposed mechanisms for the antifungal activity of NK cells, including the necessity of direct cell-fungus contact (13, 36, 37). For instance, NK cell-mediated killing of *C. neoformans* requires physical interaction (36). Similarly, our prior work has demonstrated that contact between Vγ9Vδ2 T cells and *A. fumigatus* hyphae is essential for antifungal activity (13), supporting the current findings. NK cells are known to exert cytotoxic effects via cell-to-cell contact with the tumor or virus-infected cells (38), a mechanism that ensures targeted cytotoxicity. However, conflicting data exist; one study reported that contact between NK cells and *A. fumigatus* hyphae did not influence antifungal activity (37). Notably, the antifungal effect observed in that study was lower than that observed in our study, and paradoxically, the noncontact condition yielded stronger antifungal effects. These discrepancies may be attributed to differences in NK cell stimulation protocols and XTT assay conditions.

Perforin has been implicated as a critical effector molecule in NK cell-mediated antifungal responses against *A. fumigatus* and *C. neoformans* (36, 37). As a 70 kDa pore-forming glycoprotein, perforin facilitates passive transport of ions, water, peptides, and enzymes by forming 5–20 nm pores in target membranes (39). This mechanism suggests that perforin disrupts fungal cell-membrane integrity and homeostasis. However, whether perforin can exert similar effects on fungi with robust cell walls and extracellular matrices remains uncertain, necessitating further investigation. Interestingly, NK cells retained partial antifungal activity following EGTA pretreatment, implying the incomplete inhibition of degranulation or involvement of alternative mechanisms. Advanced genome-editing approaches, such as the CRISPR-Cas9-mediated knockout of degranulation pathways, may provide deeper insights into these mechanisms.

Some studies have reported direct antifungal effects of IFN-γ on *A. fumigatus* hyphae (24, 30), although these findings remain contentious. Bouzani et al. observed approximately 30% antifungal activity at rIFN-γ concentrations exceeding 2,000 pg/mL in the XTT assay; however, we were unable to replicate this effect. Another study reported a modest 6% antifungal effect at 32 pg/mL, with no enhancement at higher concentrations (30). These marginal effects may reflect assay variability or methodological differences. Moreover, both our study and previous reports have shown that the co-culture of NK cells with *A. fumigatus*, *Mucor* spp., or *C. albicans* reduces IFN-γ secretion (31, 37, 40, 41), raising questions on the relevance of IFN-γ-mediated antifungal activity *in vivo*. Further research is warranted to elucidate the potential direct antifungal role of IFN-γ and its underlying mechanisms.

A key strength of our study is that IL-2/IL-18-expanded human NK cells exhibited comparable antifungal activity against azole-resistant and wild-type *A. fumigatus* strains. These findings highlight the potential of allogeneic NK cell-based immunotherapy as a novel treatment strategy for aspergillosis. Furthermore, our mechanistic investigations revealed that IFN-γ did not directly contribute to the antifungal activity of NK cells.

Nonetheless, this study had some limitations. First, we were unable to fully characterize the degranulation pathways involved in the antifungal response of NK cells. Second, our findings were based solely on *in vitro* experiments and do not account for the complexities of the host immune system *in vivo*. Animal models are required to validate these results. Third, all NK cells used herein were derived from healthy donors, which may not accurately reflect the immune responses of patients with pulmonary aspergillosis.

### Conclusion

This study demonstrated that cellular immunotherapy utilizing IL-2/IL-18-expanded NK cells represents a promising therapeutic approach for patients with aspergillosis caused by azole-resistant strains. Furthermore, IL-2/IL-18-expanded NK cell-based immunotherapy may offer an effective alternative treatment for immunocompromised individuals with IA, particularly in cases where conventional antifungal agents fail to achieve satisfactory outcomes.

## Data Availability

The raw data supporting the conclusions of this study are made available by the authors.

## SUPPLEMENTAL MATERIAL

Figure S1A spectrum.03372-25-s0001.TIF Flow cytometric analysis of NK cell expansion after 10-day stimulation with IL-2 and IL-18.

Figure S1B spectrum.03372-25-s0002.TIF Morphological observation of NK cell clustering after IL-2/IL-18 stimulation.

Figure S1C spectrum.03372-25-s0003.TIF Flow cytometry analysis of surface-marker expression in IL-2/IL-18-expanded NK cells.

Figure S2 spectrum.03372-25-s0004.TIF Expansion and characterization of Vγ9Vδ2 T cells from a healthy donor using tetrakis pivaloyloxymethyl 2-(thiazole-2-ylamino) ethylidene-1,1-bisphosphonate (PTA) and IL-2.

Figure S3 spectrum.03372-25-s0005.TIF Antifungal activity of supernatant from natural killer (NK) cells (1 × 107 cells per well) cultured for 24 h without fungi.

Supplemental material spectrum.03372-25-s0006.docx Expanded culture of natural killer cells and Vγ9Vδ2 T cells.

ASM does not own the copyrights to Supplemental Material that may be linked to, or accessed through, an article. The authors have granted ASM a non-exclusive, world-wide license to publish the Supplemental Material files. Please contact the corresponding author directly for reuse.
