# Supplementary figures and images for: Human natural killer cells exhibit potent antifungal activity against azole-resistant Aspergillus fumigatus and diverse filamentous fungi
Source: Microbiol Spectr. 2026 Apr 21;14(6):e03372-25. doi: 10.1128/spectrum.03372-25 (PMC13227959; doi:10.1128/spectrum.03372-25)

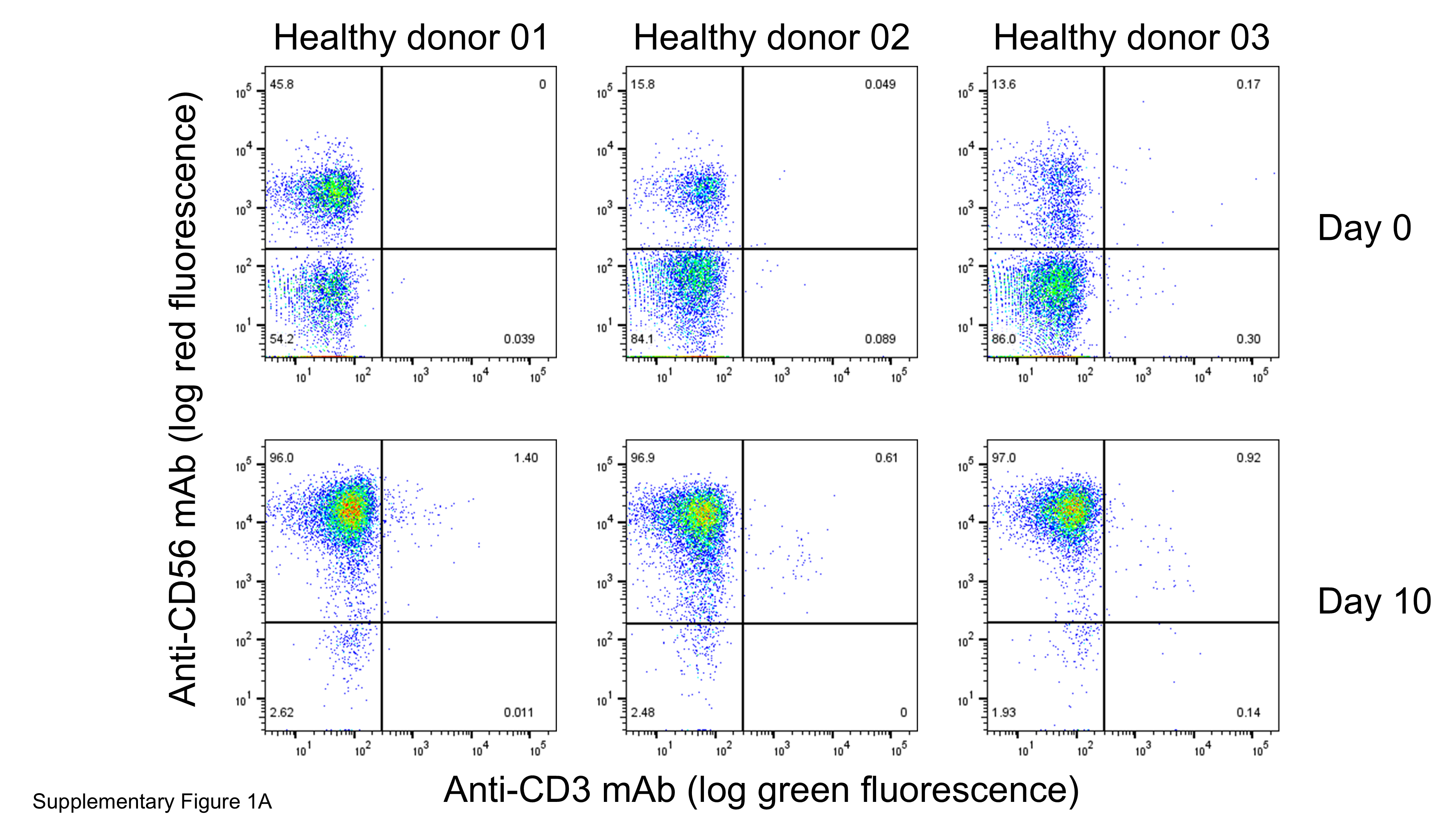

Supplement: Figure S1A — Flow cytometric analysis of NK cell expansion after 10-day stimulation with IL-2 and IL-18. [file spectrum.03372-25-s0001.tif]

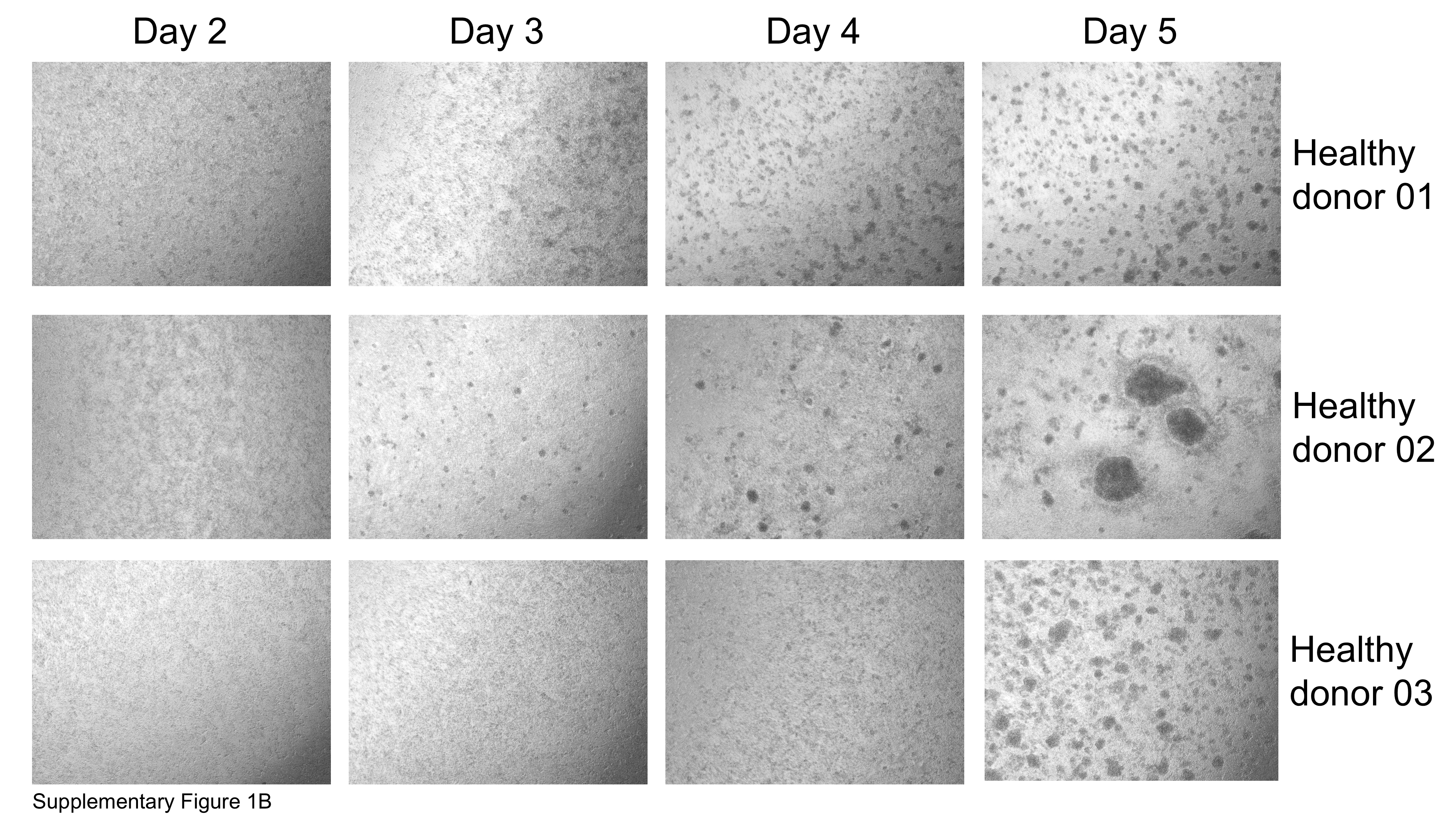

Supplement: Figure S1B — Morphological observation of NK cell clustering after IL-2/IL-18 stimulation. [file spectrum.03372-25-s0002.tif]

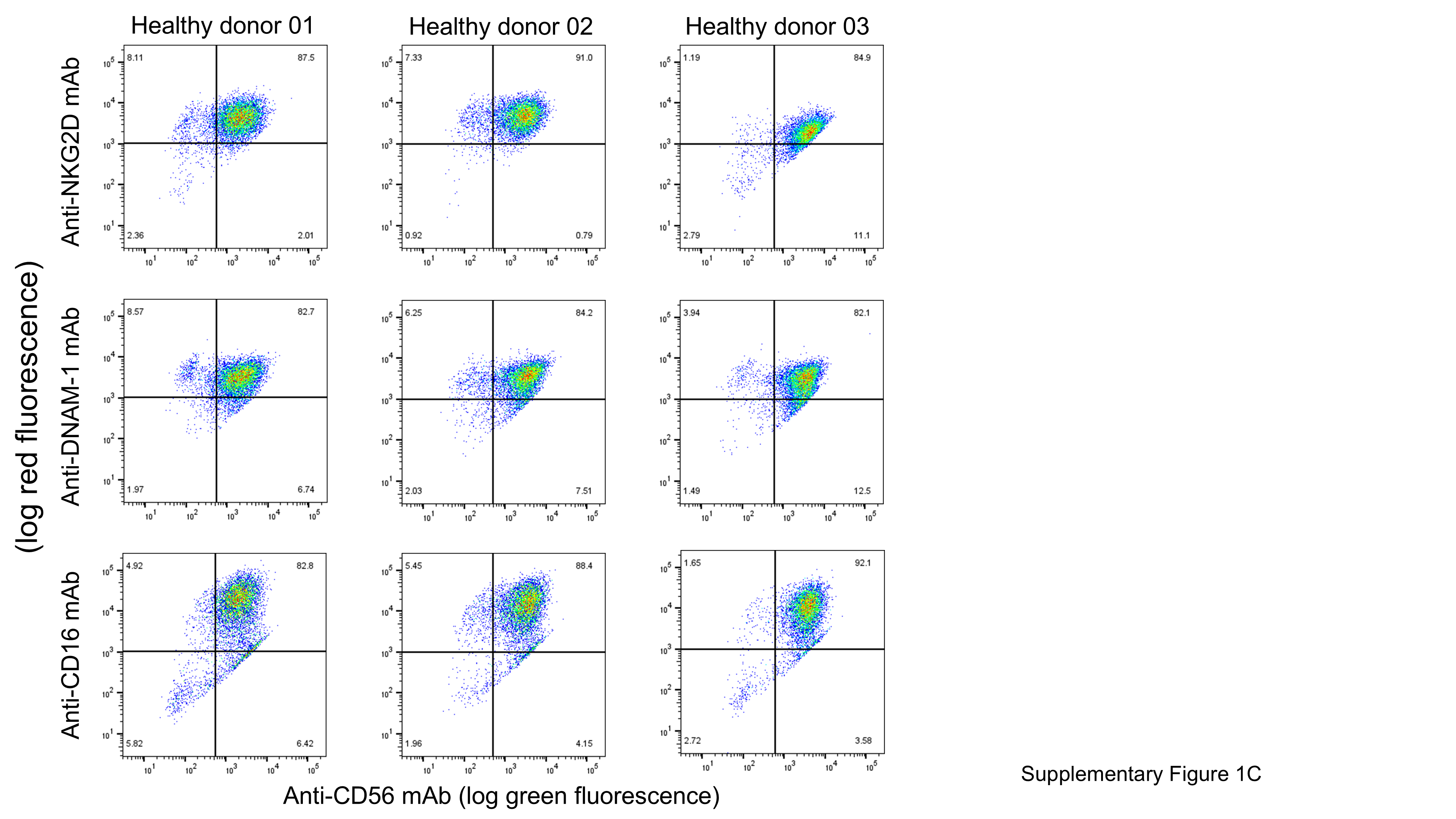

Supplement: Figure S1C — Flow cytometry analysis of surface-marker expression in IL-2/IL-18-expanded NK cells. [file spectrum.03372-25-s0003.tif]

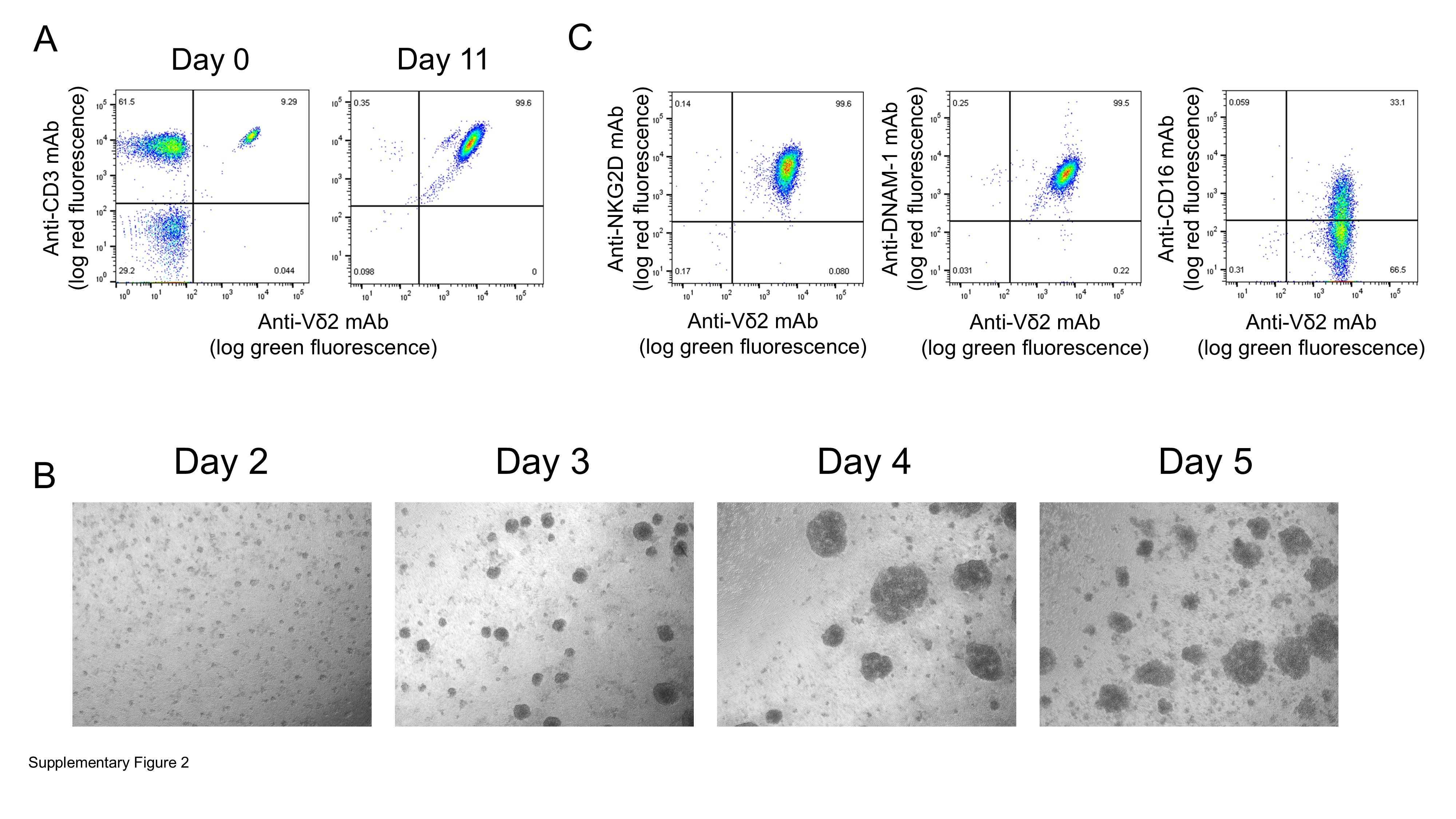

Supplement: Figure S2 — Expansion and characterization of Vγ9Vδ2 T cells from a healthy donor using tetrakis pivaloyloxymethyl 2-(thiazole-2-ylamino) ethylidene-1,1-bisphosphonate (PTA) and IL-2. [file spectrum.03372-25-s0004.tif]

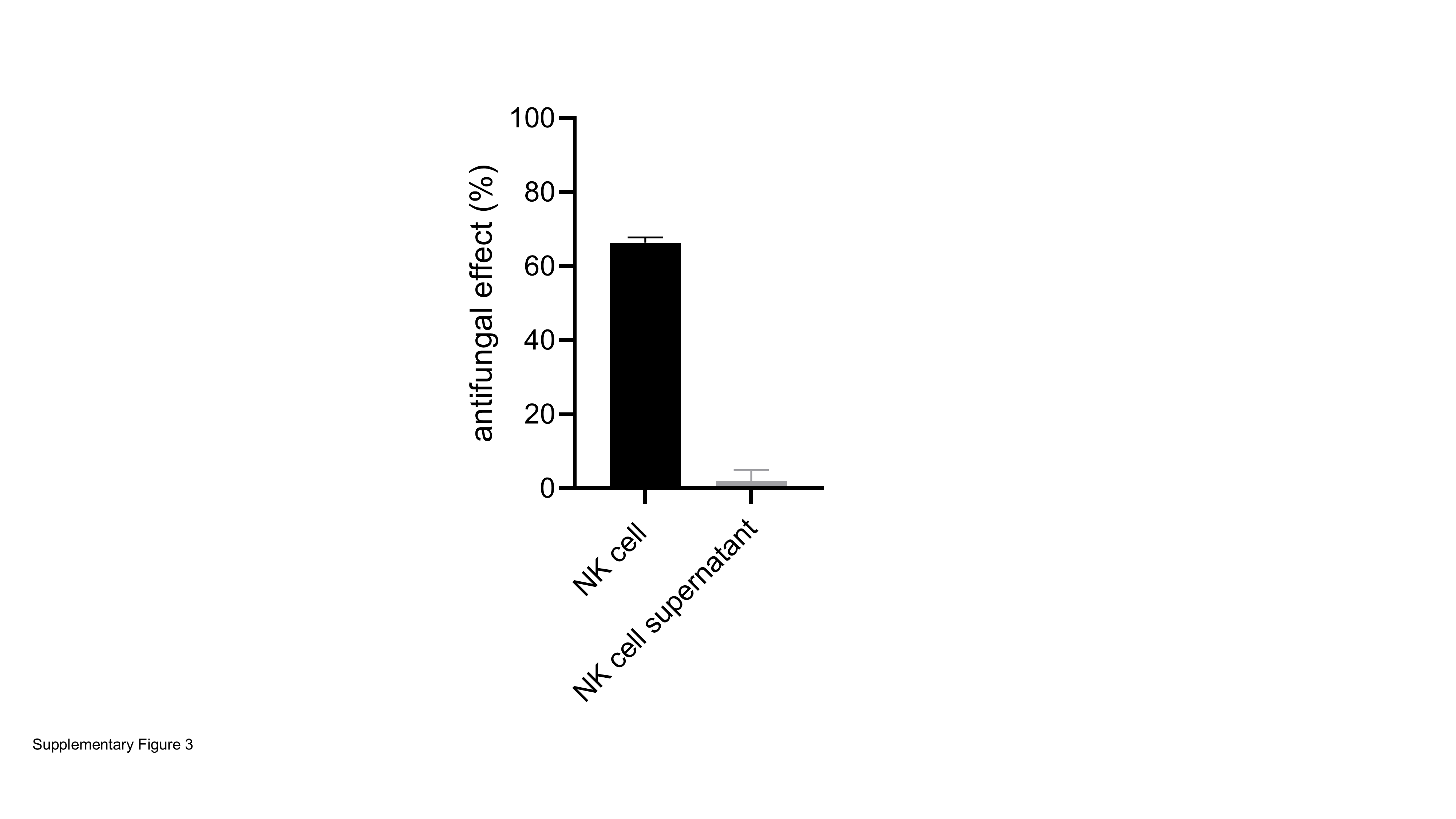

Supplement: Figure S3 — Antifungal activity of supernatant from natural killer (NK) cells (1 × 10^7^ cells per well) cultured for 24 h without fungi. [file spectrum.03372-25-s0005.tif]
