# Supplementary material for: Human natural killer cells exhibit potent antifungal activity against azole-resistant Aspergillus fumigatus and diverse filamentous fungi
Source: Microbiol Spectr. 2026 Apr 21;14(6):e03372-25. doi: 10.1128/spectrum.03372-25 (PMC13227959; doi:10.1128/spectrum.03372-25)
Supplement: Supplemental material — Expanded culture of natural killer cells and Vγ9Vδ2 T cells. [file spectrum.03372-25-s0006.docx]

**SUPPLEMENTARY MATERIAL**

**Expanded culture of natural killer (NK) cells**

Peripheral blood from healthy donors was treated with Heparin spit (Mochida Pharmaceutical, Co., Ltd., Shinjuku-ku, Tokyo, Japan; 1/100 volume) and diluted with the same volume of phosphate buffered saline (PBS) as the peripheral blood. Thereafter, 20 mL of diluted blood was mixed with 20 mL of Ficol solution (GE Healthcare BioSciences AB, Chicago, IL, USA) and centrifuged at 600 × *g* at 25°C for 30 min. After centrifugation, 15 mL of plasma containing peripheral blood mononuclear cells (PBMCs) was transferred to 50-mL centrifuge tubes and diluted with 35 mL PBS. The PBMC suspension was centrifuged at 900 × *g* for 10 min at 4°C, the supernatant was discarded, and the pellet was suspended in 13 mL PBS. The suspension was centrifuged at 600 × *g* and 4 °C for 5 min and the supernatant was discarded; the pellet was suspended in 10 mL MACS buffer (PBS containing 0.5% BSA and 2 mM EDTA) and transferred to 15-mL centrifuge tubes. PBMCs were centrifuged at 600 × *g* and 4 °C for 5 min, and the pellet was suspended in 0.8 mL MACS buffer. PBMCs were centrifuged at 600 × *g* and 4 °C for 5 min and the pellet was suspended in 0.8 mL MACS buffer. Next, 0.2 mL of anti-CD3 MACS bead antibody (Miltenyi Biotec, Auburn, CA) was added to the PBMC suspension and incubated at 4 °C for 15 min. After incubation, 10 mL MACS buffer was added to the PBMC suspension and centrifuged at 300 × *g* for 10 min at 4 °C. After centrifugation, the supernatant was discarded; the pellet was suspended in 2 mL of MACS buffer and transferred to an LD Column filled with MACS buffer. LD Columns were fixed in magnet holders (Miltenyi Biotec) and CD3-negative PBMC were collected in new 15-mL centrifuge tubes. Thereafter, 6 mL of Yssel's medium (1) was added, and CD3-negative PBMCs were centrifuged at 600 × *g* and 4 °C for 5 min. The cell suspension was adjusted in Yssel's medium supplemented with 10% human AB serum to a concentration of 2.0 × 10^6^/mL; the cell suspension was inoculated into 24-well plates at 1.5 mL per well. Cells were cultured at 37 °C with 5% CO2 for 10 days and collected on day 10. IL-2 (Shionogi Pharmaceutical Co., Ltd., Chuo-ku, Osaka, Japan) and recombinant IL-18 (Techno Suzuta Co., Ltd., Nagasaki city, Japan) were added to each well at final concentrations of 100 U/mL and 100 ng/ml respectively from day 0 to day 8. The medium was changed with new Yssel's medium on day 2. Cells were passaged daily from day 5 to day 8; from day 6, cells were cultured in RPMI 1640 medium (Merck & Co., Inc., Darmstadt, Germany) with 10% fetal calf serum (FCS) in 75 cm^2^ flasks. On day 10, cells were collected, suspended in cell preservation solution and stored overnight at -80 °C. Thereafter, cells were stored in liquid nitrogen until they were used in experiments.

**Expanded culture of Vγ9Vδ2T cells**

Similar to NK cells, PBMCs were harvested from the peripheral blood and suspended in 13 mL of PBS. PBMC suspensions were centrifuged at 600 × *g* and 4°C for 5 min, and pellets were adjusted in Yssel's medium supplemented with human AB serum to a cell concentration of 2.0 × 10^6^/mL. Tetrakis pivaloyloxymethyl 2-(thiazole-2-ylamino) ethylidene-1,1-bisphosphonate was added to Yssel's medium at a concentration of 1 µM. The cell suspension was inoculated in 24-well plates at 1.5 mL per well. Cells were incubated at 37 ℃ with 5% CO_2_ for 11 days. IL-2 was added daily from day 1 to a final concentration of 100 U/mL in each well; Yssel's medium was changed on day 2, and cells were passaged daily from day 5 to day 9. Medium was changed to RPMI 1640 medium with 10% FCS from day 6. Furthermore, 75 cm^2^ flasks were used on Day 6, and 225 cm^2^ flasks were used on day 7 to day 11. Cells were collected on day 11, suspended in cell preservation solution and stored in overnight at -80 ℃. Subsequently, cells were stored in liquid nitrogen until used in experiments.

**Flow cytometry analysis**

Cells were analyzed using a FACS Lyric flow cytometer (Becton Dickinson & Co., Franklin Lakes, NJ) and confirmed to be pure NK cells or Vγ9Vδ2T cells. FlowJo ver. 10 (FlowJo LLC, Ashland, OR) was used to analyze flow cytometer data. The used antibodies are listed below: phycoerythrin(PE)-conjugated anti-CD3 monoclonal antibodies (mAb) (BD Biosciences), CD56, NKG2D, DNAM-1,and CD16 mAbs (BioLegend, San Diego, CA) and fluorescein isothiocyanate (FITC)-conjugated anti- CD3 mAb (BD Biosciences), CD56 mAb (BioLegend), and Vδ2 mAb (Beckman Coulter Inc., Pasadena, CA).

**References**

1. Yssel H, De Vries JE, Koken M, Van Blitterswijk W, Spits H. 1984. Serum-free medium for generation and propagation of functional human cytotoxic and helper T cell clones. J Immunol Methods 72:219-27.

**Supplementary Figure Legends**

**Supplementary Figure 1**. Expansion and characterization of IL-2/IL-18-expanded natural killer (NK) cells from healthy donors

(A) Flow cytometric analysis of NK cell expansion after 10-day stimulation with IL-2 and IL-18. Cells derived from healthy donors were stained with phycoerythrin (PE)-conjugated anti-CD3 monoclonal antibody (mAb) and fluorescein isothiocyanate (FITC)-conjugated anti-CD56 mAb before and after expansion and were analyzed using a FACS Lyric flow cytometer. After expansion, the proportions of CD3 ^−^ CD56⁺ NK cells increased to 96.0%, 96.9%, and 97.0%, respectively.

(B) Morphological observation of NK cell clustering after IL-2/IL-18 stimulation. Cell aggregation was visualized using a microscope equipped with a CCD camera (Olympus Corp., Shinjuku-ku, Tokyo, Japan).

(C) Flow cytometry analysis of surface-marker expression in IL-2/IL-18-expanded NK cells. After 10 days of stimulation, cells were stained with PE-conjugated anti-NKG2D, DNAM-1, and CD16 mAbs, and FITC-conjugated anti-CD56 mAb. More than 80% NK cells expressed high levels of NKG2D, DNAM-1, and CD16.

**Supplementary Figure 2**. Expansion and characterization of Vγ9Vδ2 T cells from a healthy donor using tetrakis pivaloyloxymethyl 2-(thiazole-2-ylamino) ethylidene-1,1-bisphosphonate (PTA) and IL-2.

(A) Flow cytometric analysis of Vγ9Vδ2 T cells following an 11-day stimulation with PTA and IL-2. Cells were stained with phycoerythrin (PE)-conjugated anti-CD3 mAb and fluorescein isothiocyanate (FITC)-conjugated anti-Vδ2 monoclonal antibody (mAb) before and after expansion and were analyzed using a FACS Lyric flow cytometer. Postexpansion, the proportion of CD3⁺Vδ2⁺ T cells increased to 99.6%.

(B) Morphological observation of Vγ9Vδ2 T cell clustering following PTA/IL-2 stimulation. Cell aggregation was visualized using a microscope equipped with a CCD camera.

(C) Flow cytometric analysis of surface marker expression on PTA/IL-2-expanded Vγ9Vδ2 T cells. After 11 days of stimulation, cells were stained with PE-conjugated anti-NKG2D, DNAM-1, or CD16 mAbs, and FITC-conjugated anti-Vδ2 mAb. Flow cytometry revealed that nearly all expanded Vγ9Vδ2 T cells expressed high levels of NKG2D and DNAM-1.

**Supplementary Figure 3.** Antifungal activity of supernatant from natural killer (NK) cells (1 × 10^7^ cells per well) cultured for 24 h without fungi. Bar graph shows the mean and standard deviation of technical replicates (*n*=8). Data are representative of at least three independent experiments.
